# The Spanish Labor Market: A Gender Approach

**DOI:** 10.3390/ijerph18052742

**Published:** 2021-03-08

**Authors:** Mª Genoveva Dancausa Millán, Mª Genoveva Millán Vázquez de la Torre, Ricardo Hernández Rojas, Juan Antonio Jimber del Río

**Affiliations:** 1 Department of Statistics, Córdoba University, 14071 Córdoba, Spain; z62damim@uco.es; 2 Department Quantitative Methods, Universidad Loyola Andalucía, 14004 Córdoba, Spain; 3 Department of Agriculture Economics, Finance Accounting, Córdoba University, 14071 Córdoba, Spain; ricardo.hernandez@uco.es (R.H.R.); jjimber@uco.es (J.A.J.d.R.)

**Keywords:** labor market, wage gap, glass ceiling, Spain, ARIMA

## Abstract

The massive incorporation of Spanish women into the labor market is a phenomenon that began in the second half of the 20th century, being many the obstacles that this group has had to overcome to reach the current situation, where getting a job can be an achievement that, in many cases, does not correspond to the capacity and academic training of the worker, creating a labor and economic imbalance (the cost in training is not rewarded with the work done). In this work, the Spanish labor market was analyzed through the labor force survey (EPA) from a gender perspective, demonstrating the existing inequalities at the labor level, both of employment and unemployment rates, and of jobs where the glass ceiling is evident and of economic remuneration where the salary gap continues to be important. In addition, through an ARIMA model, the evolution of the number of Spanish working women was analyzed, and how the economic crisis of 2009 and the sanitaria have affected their employment in the various crises (COVID-19). Measures to solve the problem as well as laws and active policies in favor of the creation of female jobs and a greater awareness of empowerment on the part of the female collective are proposed.

## 1. Introduction

From the 1960s, when Spanish women began to play a relevant part in the labor market, until now, there have been many challenges and obstacles that they have had to overcome to try and acquire a similar role to that of men in all areas, especially in the work area. This has not been translated into results as effectively as has been expected in comparison with the effort made. This is due to a great part to the major labor inequalities (access to jobs) and remunerations (wage gap) between women and men [1,2,3].

Analyzing the different economic theories on the labor market, in accordance with the postulates of one of them, neoclassical theory, which supposes that the labor markets function efficiently, entrepreneurs maximizing their profits and workers optimizing their work incomes, wages will be according to the marginal product of work. Based on this argument, if women are participating increasingly more in the labor market, if they have a greater educational level, and more work experience, this would induce a significant restructuration of their remunerations and their preferences with respect to the type of work that they are employed in or which is offered to them. However, this is in reality not being fulfilled in a large part of the labor market, as is demonstrated by the studies done in this field [4,5,6,7,8,9,10,11,12,13]. These indicate that wage discrimination and occupational segregation still exist in the 21st century.

However, this phenomenon not only affects Spain but all countries to a greater or lesser extent. For example, in Argentina [14,15], the wages women earn for their work is less than that of men due to a more precarious employability. A smaller proportion of female workers access managerial positions, although they have better educational levels. This even takes place in highly feminized activities. The gender wage gap is the result of an inequitable distribution of the educational capital. This explains why women access quality employment less and are worse remunerated than men. Since the 1970s, attempts have been made to quantify wage discrimination and wage differences with a gender approach. Studies in the United States [16,17]. Mexico [18,19,20]. and Uruguay [21] stand out. The latter study tried to quantify via regressions the behavior of the gap between male and female wages through wage distribution.

In Spain, in the study and analysis of the labor market from the gender perspective has become in itself a topic of evident and insurmountable importance, taking into account the procedure of Corporate Social Responsibility (CSR) when knowing professional paths and profiles [22,23,24,25,26]. Women, half of human capital, have irrupted into the labor market, revolutionizing this area, but they have not obtained as positive a response from it as expected, given that there is a strong difference in the employment and unemployment rates between both groups [27,28]. However, although a lot remains to be done, the process of change is unstoppable.

This work aims to carry out an investigation of the Spanish labor market placing a special emphasis on women to determine how this market has evolved and the situation of the female Spanish worker. We would thus be able to respond to questions such as the following: Has the number of jobs increased at the expense of labor precariousness? Where is the line that separates a decent job and the creation of underemployment that decreases the figures of the unemployed? According to Palomino [29], when there is a restructuring of the work markets that affects the condition of the employees, we are facing a situation of work precariousness. When there is a deterioration in the work conditions or in the establishing of a labor relation in not very favorable conditions for the worker, the labor precariousness is very clear [30,31]. This circumstance is affecting women in Spain, as women represent the immense majority of part-time and temporary contracts and therefore their wages are lower [32].

This research analyzes the Spanish government’s employment promotion measures and how they have impacted the women’s labor market (employment rates, unemployment, jobs, and the wage gap and glass ceiling), through information provided by the Labour Force Survey (EPA), as well as how women’s employment has evolved over the past 12 years (2008–2020) through an ARIMA model. There are no studies that model the Spanish women’s market, which is the originality of this research.

## 2. Evolution of the Labor Market in Spain. Employment Strategies

This research was based on Hypothesis H_1_ of the labor inequality of working women in the economic aspect (wage gap) and H_2_ job inequality (glass ceiling). To corroborate these hypotheses, a descriptive analysis and an ARIMA model were developed.

Spain is one of the 27 countries which make up the European Union (EU) and, since it joined in 1986, many of its labor policies have followed EU guidelines, as there is a free circulation of workers. Among these, the Stability and Growth Pact (SGP) and European Strategy 2020 stand out. These tackle how to achieve economic and growth recuperation in the euro zone. Within the SGP (2011), in the employment context, specific orientations are offered for the employment policies of member countries, priority areas are established where these countries must act, and structural reforms which must be undertaken to: (1) make a more attractive market; (2) reform the pensions system; (3) foster the reintegration of the unemployed into the labor market; and (4) foster the balance between labor security and flexibility.

The aim of these reforms is to decrease the number of unemployed in the EU—more than 15.9 million in 2020, of whom a fifth (3.47 million) correspond to Spain. The short-term economic expectations forecast a fast recuperation of the labor market if the public powers drive the creation of employment. In 2012, the European Commission presented a packet of specific measures to foster the recuperation of employment in Europe, based on national policies which can stimulate the labor markets. Among the recommendations is for the states to mobilize all the existing resources for the creation of employment, as not everything is public spending. The collective negotiation or investments which assure a return in costs are elements to bear in mind to guarantee more and better quality employment. Flexibility must be linked to security and one can act with reforms in labor legislations and incentives to reinforce the connection between education and work. Some of these proposals are:

- Subventions for contracting that generate new jobs
- Transfers of the labor taxation to environmental taxes
- Support for self-employment
- Impose internal flexibility to reduce employment insecurity and tax costs
- Establish decent and sustainable wages and avoid the traps of low wages
- Foster training to adapt the workers’ capacities to the needs of the labor market

**Figure 1:**
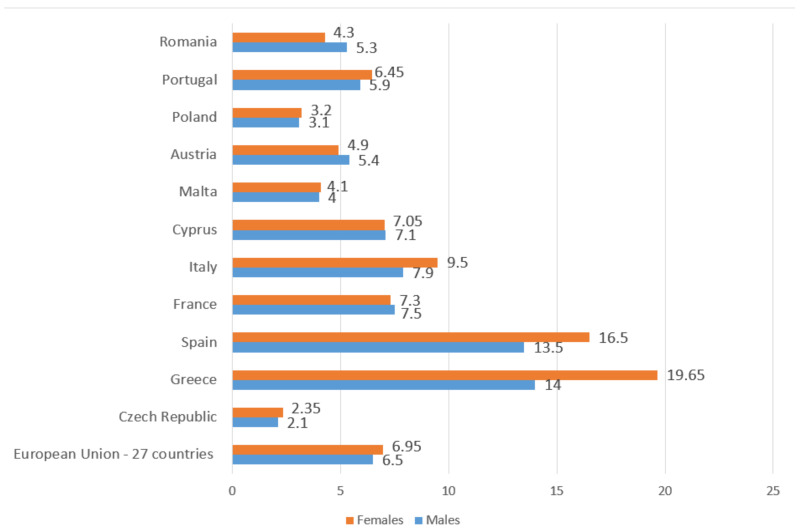
Male and female unemployment rate, in percentage (first and second quarter of 2020). Source: Own elaboration from data of Eurostar [33].

This packet of measures has been translated into a decrease in the unemployment rate from 10.7% in 2013 to 6.7% in 2020. This evolution for women and men has been from 10.8% to 6.9% and from 10.6% to 6.5%, respectively, for this period of time. However, the behavior has not been the same in all countries. Figure 1 reflects the six countries with the greatest and lowest female unemployment rates in the first and second quarter of 2020. The high rates of female unemployment in Mediterranean countries standing out, such as Greece (19.6%) and Spain (16.5%), which are triple and double the European average (6.9%). The counterpart is Central European countries, such as the Czech Republic and Poland with the lowest rates (2.3% and 3.1%, respectively), which equate to a third of the European average (considering these countries as having full employment as their unemployment rate is less than 5.2%).

**Figure 2:**
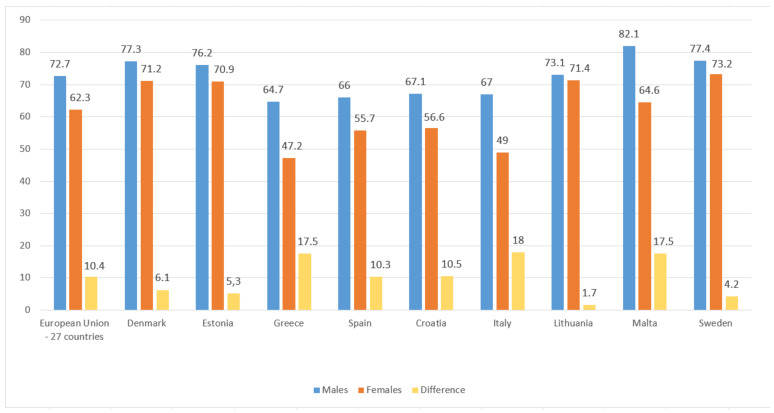
Male and female employment rate, in percentage (first and second quarter of 2020). Source: Own elaboration from data of Eurostar [33].

As to the female employment rate, the Mediterranean countries are again those which have the lowest rate, Greece being prominent with 47.2%, followed by Italy with 49%, and in third place Spain with 55.7%. On the contrary, the countries of North and Central Europe are those which have the highest rates. At the head is Sweden with 73.2%, Lithuania with 71.4%, and Denmark with 71.2%. Figure 2 shows the enormous difference between the male and female employment rates, Italy surpassing more than 18% followed by Greece and Malta with 17.5%. These data undoubtedly bring to light the labor gap between men and women.

Although in Spain the participation rate of women in economic life has increased in recent years (more than 1.9% in six years, from 53.8% in July 2014 to 55.7% in July 2020), it remains poor (55.7% compared to 66% for men, Spanish National Institute of Statistics (INE), July 2017). The rate of Spanish activity is one of the lowest of the EU and for example it has not reached the active population rate of Sweden in 1995. The demographic data demonstrate that, in the countries of North Europe, and specifically in the Scandinavian countries, which have a lower female population, there is a greater number of active women than in the countries of South Europe.

Centering ourselves on the evolution that has happened in Spain, it is worth highlighting that in 1960 there were less than 2.5 million women working outside the home. However, in the 1960s, the massive increase of their participation in the labor market began. This reached 8.5 million in the second quarter of 2020, having risen 2.4% with respect to the same quarter of 2016. This growth figure is not very high. This is because of the crisis that the country has endured since 2008, also aggravated by COVID-19 (2019–2020), which has resulted in an increase of the number of unemployed (both men and women). This reached 6 million in 2013. Through the diverse measures adopted by the national government and those of the regional communities, an attempt has been made to reduce this figure. At the moment (second quarter of 2020), there are less than 3.3 million unemployed, this being an indicator of the country’s economic recuperation. Nevertheless, the group of women, being more sensitive than that of men, has been more affected by the fluctuations in the economy [34,35].

In 2019, and as an effect of the measures addressed in the labor market, unemployment went down to 3.2 million, of whom 1.7 million are women and 1.5 million are men. However, while the number of unemployed men went down with respect to 2018 by 231,562, the number of women unemployed did so to a lesser extent, 146,265. Similarly, those employed grow by 66,913 in the group of men compared to 153,487 in the group of women. Hence, the measures of fostering contracting have not been as completely effective as forecasted in the group of women (Table 1).

To achieve the aims of increasing female employment and decreasing unemployment in this group, successive Spanish governments have elaborated, within their employment policies and the framework of the European Employment Strategy, various legal measures to promote the incorporation of women into work.

Various factors influenced this increase, among which are: (1) a greater educational level; (2) new household appliances [37]; (3) the generalization of contraceptives; (4) the growth of the services sector; and (5) the awareness of those interested in acquiring financial autonomy (according to a study done in 1999 [38], for 33% of Andalusian women to be successful means being economically independent, while for 29.2% it means having children). In the framework of the European Strategy for Employment, different legal measures favor the entry of women into the workplace. The last measure has been the Strategy of Activation for Employment 2017–2020, which is oriented toward six basic objectives:

1. Create employment: The central aim of economic policy is to create employment, placing emphasis on female employment. It aims to raise the participation in the labor market and reduce unemployment by achieving an employment rate of 74% for the population between 20 and 64 years old in the horizon 2020, with a sub-objective of a female employment rate for the same age group of 68.5% in 2020. In this period, not only the total volume of employment will increase, but also the degree of qualification of the workforce, due to the new cohorts that will enter into the labor market being more qualified than those who leave it. The intermediate objective (2015) was to reach an employment rate of 66%. However, in 2014, this was 45.04%, 3.36% lower than in 2010, and in 2017 it is 63.9%, 2.1% below the aim for 2015 and 10.1% from the objective for 2020. The group of women is 10.4% from achieving the 2020 objective of 68.5%. This will be difficult to reach if more active policy measures are not taken.
2. Reduce the seasonality of contracts. Currently, a fourth of employment is subject to a system of unstable contracts, with an excessive turnover. This limits the improvements of firms’ productivity. To combat this trend, this objective establishes its reduction.
3. Reinforce part-time work and firms’ internal flexibility. The lack of an appropriate application of internal flexibility in firms has meant that the adjustments have been concentrated in employment. This is why this objective aims for the adjustments in the labor market not to fall necessarily on employment, via the use of other mechanisms such as the reduction of working hours.
4. Improve and adapt professional competences to the needs of the market. The lack of the appropriateness of professional competences for the profiles required is one of the main obstacles which hinder the efficient functioning of the labor market. Through this objective, it is sought to foster the improvement of education and learning, adapting them to a changing labor market and spanning from the reduction of school dropout to the increase of the percentage of the adult population who receives training for employment.
5. Promote a fast and appropriate reintegration of people into the labor market. Active employment policies must facilitate access to an appropriate job as quickly as possible, especially in the case of the groups which merit particular attention in this strategy. This aim is monitored mainly by noting the long-term unemployment rates in each of these groups and evaluating the effect that investment in active employment policies can have.
6. Promote gender equality in the labor market. The difficulties in the access to and promotion of women in employment in conditions of equality are revealed in the lower rate of female employment. This is why it is necessary to promote gender equality in the labor market. This objective proposes to progressively reduce the gap that currently exists between the female employment rate (58.1%) and that of males (69.6%), as well as the one between the average hourly wages of men and women.

The monitoring of these six objectives of employment policy is being carried out through a set of appropriate indicators. The value of these indicators reflects the result of a much broader set of social and economic policies, designed according to the National Program of Reforms.

Table 2 presents this set of indicators. Its evolution will provide information about the degree of achievement of each of the six objectives of employment policy.

Although the horizon is set for 2020, the data of intermediate years are being analyzed. Unemployment is being reduced but not at the speed forecasted. Relevant corrections of the measures will have to be done, or some additional ones will need to be created to reactivate the market more and influence more gender equality, as the female market is the one which is advancing at a lower speed despite two of the six aims directly involving this group.

## 3. Structure of the Spanish Labor Market

When examining the Spanish labor market, it is easy to obtain an answer to the question: Why do different Spanish governments have among their goals to foster female employment and achieve equal work and wages conditions for this group? The answer is the enormous imbalance between men and women in labor matters. Nevertheless, since the 1960s, the greater participation of women in different labor areas has been a social fact of particular importance. The increase of the active female population and its entry into the labor market has had a great repercussion, bringing about evident advances of women at the social and cultural level. The stages that the society has developed to accept women in the labor area can be specified as follows:

(1) Awareness of Spanish women that they have to join the workforce. The modification of the role of women within the family structure stands out (not only as the carer and safeguard of family harmony: they are now income suppliers), contributing an important part of the incomes of the family unit by remunerated work outside the home and obtaining professional development and careers.
  Change in the mentality of society, especially in men. There are not specific tasks for women and for men. To share housework and avoid the double workday (remunerated work outside the home and unpaid work at home), a more equitable distribution of housework between men and women has enabled the latter to have time to work outside the home. Men are now not the ones in charge of sustaining the family and woman the ones taking care of it. It is increasingly more considered that both men and women—the two-headed home—take on the task of providing incomes in the home. Likewise, men are doing housework. A few decades ago, this would have been unthinkable as it was associated with females. This advance in society has also been accompanied by a rejection of everything referring to tasks.
(2) From the 1940s until the beginning of democracy (1975), few women had a remunerated job outside the home. A determinant variable was the marital status, as more than 80% of Spanish women workers in these decades were single. When they got married, the immense majority (middle- and upper-class women) left their jobs and professional careers to take care of their husband and children. The role of wife and mother prevailed over that of personal realization. If a married woman worked outside the home, she was looked upon with disapproval from a social point of view. It was supposed that her husband (provider of family incomes) could not maintain her, wounding in part the male pride of the period, as these men did not have the capacity to feed and sustain a family, which can be identified as specific to a single sex. Phrases that in the 1960s were very common in Spanish homes, such as “mopping up is a woman’s job”, are now rejected by society and considered as chauvinist. It is also more common to see television commercials of household products announced for men. That is, advertising marketing has changed and is aimed at the two groups. It is a question of an evolution toward equality where phrases such as the one mentioned or “this is a man’s thing” are considered discriminatory and frowned upon by the current Spanish society.
(3) Desire of women to develop a professional career, investing in training. Spanish women do not settle for working; they want a job which fulfills them as a person and is in accordance with the training acquired.
(4) Eliminate or palliate the imbalances in the labor market through active policies of fostering employment that benefit women as they are more disadvantaged from an economic (wage gap) and social (glass ceiling) point of view.

Currently, the first three stages are in the phase of maturing. For the fourth phase, we will have to wait for the results of the measures adopted, although in the short term they have not been as efficient as was hoped.

Nonetheless, the advance of Spanish society has allowed the entry of women into the labor market to be one of the most far-reaching phenomena of recent history. Although this is an unstoppable fact, one must analyze if the work that Spanish women do is quality work and if they find themselves in equal conditions to access the labor market as men. To do so, diverse variables must be analyzed, such as the type of working hours which women do, the sort of contract, the position which they have, training, etc. These variables would permit an evaluation of the Spanish female labor market.

**Figure 3:**
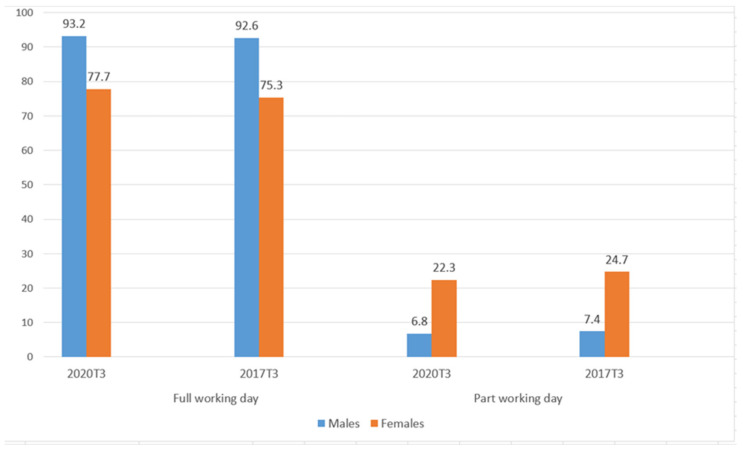
Working day by sex, in percentage (third quarter of 2017–2020). Source: Own elaboration from data of the Spanish National Statistics Institute (INE, (data free use)) [36].

Figure 3, the working day (full or part time) according to gender in Spain, highlights that the part-time working day is triple in women (22.3%) than in men (6.8%). Spanish women find part-time employment to reconcile family life, their salary being an income supplement to those of the family unit. It must be stressed that more than 93% of men have a complete working day compared to 77.7% of women. Therefore, men have greater economic stability. Even if both groups have the same work position, as the working day is shorter for women, the wages are lower.

**Figure 4:**
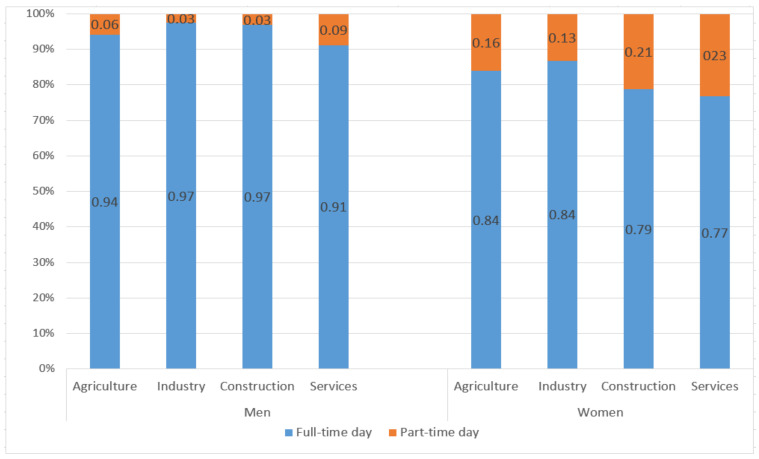
Working day by sex and activity sectors, in percentage (third quarter of 2020). Source: Own elaboration from data of the Spanish National Statistics Institute (INE, (data free use)) [36].

If we analyze the different sectors of economic activity (Figure 4), it is noted that the percentage of the complete working day of permanent contracts of males is higher than that of females in all cases. The part-time working day is highest in women in the services sector (23%). Of the 1.9 million part-time workers, 447,387 are employed in the services sector. On the contrary, fewer than one million men have part-time contracts (710,888). The services sector is the only feminized sector—the group of women workers is higher than that of men (6.8 million compared to 5.4 million).

Another clear indicator of employment precariousness, as well as the type of working day, is the contract’s seasonality (Table 3). In all sectors except for construction, men have higher permanent contract rates than women. In the construction sector, which is an activity area clearly dominated by men, only 73.4 thousand women have a permanent contract compared to 505.5 thousand men. In this sector, the great majority of women are in highly qualified categories as architects or engineers. However, 16.4% are employed with a temporary contract [39]. They corroborate the inequality assumptions raised.

To decrease female job precariousness by increasing the rates of permanent work will give women more stability in employment and the possibility of trienniums for long service. This would increase their wages and thus decrease the wage gap that exists between men and women.

**Figure 5:**
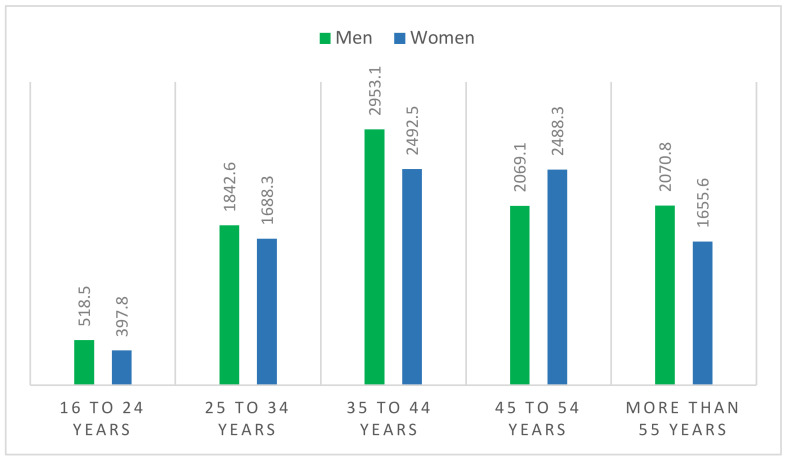
Employment by sex and age (thousands of people, 2020). Source: Own elaboration from data of the Spanish National Statistics Institute (INE) [36].

Regarding the age of Spanish employed workers (Figure 5), it stands out that the majority are 35–44 years old, this being the range where we find 29.2% of the Spanish labor force, while the most unprotected age group is that of young people who wish to obtain their first job (16–24 years old age group). Salaries increase every three years of active service in a firm or organism.

Analyzing the unemployment rates according to the level of studies (Table 4), it is noted that, as Spanish women become more prepared academically compared to men, their employment rates are higher, there being a gap of 2.748% in higher education. If they are academically trained, why is there an imbalance in the market? Does the market not socially accept Spanish female workers even though they academically have enough training? Notwithstanding, in absolute values, the entry of women into the labor market has been growing in the last decades, although not at the rate that it should.

Until now, we describe the quantitative advance. The following question is whether, in parallel to these changes of the social environment that have favored the entry of women into the labor world, an important evolution in their qualification has also taken place.

Regarding the training of women, the quantitative increase of female workers has led to a parallel development of their qualitative weight in the labor market. However, we are witnessing a change of direction in this sense.

If employment and wage discrimination has been encouraged, this has been precisely because women have traditionally entered sectors considered as feminine. The strength of tradition and the social majority of thought about the capacity of the female sex have directed women toward specific professions, pigeonholing them and restricting them.

However, there are various facts that have increasingly more weight and which are favoring the qualitative leap. This is the massive access of women to higher education, the increase of specialization and permanent training, and the increase of experience that women are achieving.

In the last decades, the access of women to higher education has been very high. Today, they are more than half the students in colleges. Only in technical colleges do they continue being a minority, especially in engineering and technology (INE, Statistics of university teaching in Spain).

Table 5 shows that 45.6% of the people who are currently working in Spain have studied in universities (more than 8 million, third quarter of 2020), and within this group women represent 51.8% (4.5 million compared to 4.2 million men). One has to ask if their job positions are in accordance with their qualifications.

According to the INE’s survey of the active population, the employment rate per studies completed has risen between 2000 and the second quarter of 2013 by 3.7% for women with primary studies, 11.2% for women with secondary education, and 6.3% for women with higher education. Nevertheless, it is worth highlighting that in the levels of higher education the number of women employed surpasses by 323.8 thousand people that of men (third quarter of 2020). However, one must ask if they really have a job position in accordance with their level of studies or are underemployed.

Our attention is drawn to the services sector having been the greatest generator of female employment. Within this, health is the activity sector that has experienced an important process of feminization in the last years. This is along with a qualitative change, where women have been getting jobs of greater professional qualification.

There are now few professions which women do not engage in, but it is important to highlight that that they mainly have positions that enable them to reconcile their professional and family lives. Thus, 70% of Spanish female workers are concentrated mainly in five sectors: civil servants, employees of firms, domestic workers, non-qualified industrial workers, and teachers and health-related professions (nurses and doctors). Undoubtedly, for this reason, there are few Spanish women who have an industrial or scientific career. It is also infrequent for women to get jobs as executives or have senior positions in firms. This is demonstrated by the fact that, in the management teams of the 1000 most important firms in Spain, only 3.25% of the posts are occupied by women.

According to Millán [40], there are more university women with better records than men but who study careers related with social and health sciences, there being a low percentage pursuing technical studies (engineering and architecture).

By activity sectors (Table 6), according to the same source previously mentioned, the employed population does not have a similar structure according to the person’s sex. Hence, women surpass men by more than 1.7% in domestic staff (2.3% compared to 0.6% of men), 4% in the sector of education (8.1% of employed women are in education compared to 3.8% of men), and 10.9% in the health sector (13.6% of employed women work in health-related jobs compared to 3.6% of men). On the contrary, there are other sectors where the presence of men is a majority, such as in construction (10.6% of men are employed in construction compared to 1.1% of women) or the manufacturing industry (15.3% of men compared to 6.8% of women). This indicates that specific sectors have the name of women, there being a strong employment segregation [41].

The profile of woman workers is also changing according to their professional situation. The participation of women in the public sector has increased from 29.2% in 1982 to 55.4% in the second quarter of 2020, while that of wage-earning women in the private sector has gone from 25.80% to 43.50%, according to data of the INE, second quarter of 2020.

However, there is more. Another gap fact in the history of female labor is in the data of the second quarter of 2020. Of the total of people who have jobs as directors or managers of firms (4.6% of those employed), 30.6% are women, men being the ones who predominate in executive positions and, therefore, the “glass ceiling” is still in evidence, despite the NOW (New Opportunities for Women) programs, whose objective is, faced with the difficulty of the professional promotion of women to positions of responsibility, to foster women accessing executive positions through diverse actions. This problem, which prevents women from having positions of responsibility in spite of their training, not only affects Spanish working women. Accordingly, it has been analyzed by numerous authors since the 1980s, either from invisible barrier workwise that prevents women from continuing to advance in their professional careers, despite that, according to Burin (2008), there are neither laws nor codes that say that “women cannot occupy these job posts, in practice there are family and social codes which tacitly impose this limitation on the gender”. The country perspective, such as in the United States [42], Ireland [43], India [44], Spain [45,46], Uruguay [47], Chile [48], Switzerland and Albania [49], Japan [50], and Ukraine [51], or in labor fields, such as in the sector of stockbrokers [52], medicine [53,54,55], advertising [56,57], education [58,59], tourism [60], communication [61], and politics [62]. This glass ceiling therefore exists in all sectors and especially in those in which there is a greater rate of feminization, such as education and health.

As indicated by the study of Sarrió [63], organizational culture, gender stereotypes, and women assuming family responsibilities as a “duty” associated with their gender role are the main hurdles in their professional promotion. Therefore, Spanish women can be better trained academically (a greater percentage of women with university studies: 44.0%, compared to 36.6% of men), but the indivisible line of direction is still in part delimited by sex instead of academic training. Nonetheless, women have a greater percentage of participation in technical, professional, scientific, and intellectual employment (51.7% in the public sector compared to 32.6% of men, a figure that drops to 12.9% in the private sector, although it is still higher than that of males at 11.12%). For now, Spanish women will be technicians but will have problems climbing to managerial positions despite their personal, professional, and academic worth.

## 4. The “Steel Ceiling” or Wage Gap

As a consequence of low qualified jobs, more precarious employment due to its seasonality, working days more reduced than those of men, and managerial positions that are difficult to access, women’s salaries are affected. Therefore, Spanish women workers find another barrier that is difficult to flank, which is the wage gap or “steel ceiling”. The European Commission in their 2012 annual report specified that, in certain cases, women and men do not receive the same wages despite doing the same job or a job of equal value. One of the main reasons is because they do different jobs in different sectors of economic activity: approximately 80% of the workers in health and social work are women. These, in turn, are the sectors that have lower wages and are compatible with their family responsibilities.

Women and men have different accesses to professional development and training in their careers. Women are underrepresented in both politics and the economy. Only 32% of scientists and engineers in Europe are women, and women only have access to 15.8% of managerial positions on the boards of directors of the major firms listed on the stock exchange in Europe, where in only 3.3% of the cases are they presidents.

This female wage gap increases even more when women have children and have part-time jobs. In 2010, the women employed in Europe with dependent children were only 64.7% compared to 89.7% of men with children. The report of the International Labor Organization (2020) puts the wage gap for 2020 at 14.1% and proposes that, to decrease it, there must be an increase in social investment in basic infrastructure and in measures aimed at reconciling labor and family commitments, to assure that the providing of care is evaluated so that the gender perspective is taken into account, centering on public services and achieving quality and affordable services of childcare and other social services as a universal right.

To reduce the wage gap would help to reduce the levels of poverty and would increase the incomes of women during their entire life. Because women have increasingly greater expectations about their professional lives, if firms wish to attract the best talent, equality in the workplace is an obligation. Many women have underused skills and capacities. Firms that create quality plans at work are also the best to work in, regardless of the worker’s gender. Gender equality is fundamental to achieve not only employment growth but also competitiveness and economic recuperation.

However, this problem does not only affect Spain. In Europe, women earn on average 14.1% less than men—in Spain the figure is around 11.9% [64].

To avoid these imbalances, the European Commission means to close the wage gap through legislative and non-legislative measures with the “Strategy engagement for gender equality 2016–2019” and establish actions in five areas: economy and the labor market, equal wages, equality in senior positions, eliminate gender violence, and foster equality in all EU countries. Gender equality and to make better use of women’s talents and skills are basic needs to reduce the wage gap and to achieve the objectives of the Strategy Europe 2020; this is materialized in attaining an employment of 75% for all women between 20 and 64 years old in Europe.

At the Spanish level, and according to the INE’s data, the wage gap between men and women has increased. Women earn an average of 22.55% less per year (approximately 5744 euros less) for work of the same value. This inequality is mainly found in the private sector where these differences increase because men get wage premiums for availability, weekends and nights. Of course, there are also differences in regions, Navarre (25.15%), Aragón (25.41%), and Asturias (25.31%) being where there is more imbalance and the Canary Islands (16.13%), the Balearic Islands (14.66%), and Extremadura (14%) where there is less.

A study called “Determinants of the gender wage gap in Spain”, done by the High Council of Chambers in collaboration with the Ministry of Health, Social Service and Equality, underlines the wage gap between men and women and places it at an average of 20%.

According to this report, the greater is the responsibility, the higher is the wage gap, and it speaks of a gap of up to 33.2% in top management positions, while the average of the wage gap in administrative positions is 29.9%, in technical positions is 20.3%, and where there is no responsibility it is 11.9%.

Meritocracy also runs into another blunt reality. The report indicates that the difference increases if the training of the person is greater. The difference in people without studies is 17.7%, with obligatory secondary education is 25.3%, with a formal degree of professional training is 26.7%, and with a university degree is 30.3%.

According to the study’s conclusions, it is stated that in no case do women get better remuneration than men for personal, business, geographical, or competitive conditioning factors.

It is also concluded that 53% of the difference for women with a permanent salary compared to their male companions is only attributable to discriminatory factors; women earn 11% less than men and, in the case of wage inequality, the difference is 19%.

It is evident that, despite basic legislation being necessary, one cannot move backward. It is necessary to continue advancing, developing, and, especially, applying legislation that eradicates gender discrimination and equips women with tools to attain effective equality of conditions. The pandemic has put a brake on this equality and the cuts in rights have caused a regression in women’s quality of life.

As a result, Spanish women must work 82 days more per year (according to the latest data of the INE, 2020), given that women’s work is still considered as something “secondary and supplementary” to family incomes. Forty-seven percent of female workers get a salary of less than 15,000 euros per year. To avoid these imbalances, labor unions propose positive action clauses for promotion and training, as well as negotiating plans and measures in firms and combating the stereotypes and gender roles that in general condition the professional participation of women and reproduce the sexual division of work.

However, discrimination is not only translated at a labor level. The Global Gender Gap Report 2019, done annually by the World Economic Forum (WEF) to evaluate the countries according to the degree of social balance which they have achieved between the sexes, gathers information from 135 countries (93% of the world population), qualifying the countries according to their capacity to close “the gender gap” based on four areas considered to be key: access to health, education, political participation, and economic equality. Spain has gone up five positions in this ranking (from 12th to 7th, during 2012–2019). This rise in its qualification is due to the increase in the number of women in positions of ministerial responsibility. In the rankings, a score of one reflects total equality in this report. No country gets it. The best score is for Iceland (0.87) and the worst for Yemen (0.54). Spain obtains an average result of 0.77. By areas, Spain achieves its best mark, 0.99, in “educational achievements” (38 in the classification), followed by 0.97 in “health and mortality” (34 in the ranking), 0.64 in “economic participation and opportunities” (75), and 0.28 in “political influence” (27), accepting Hypothesis H_2_ of inequality in the occupation of jobs (glass roof), as women have higher levels of studies but occupy lower positions than men.

Therefore, the measures to combat inequality must not only have to do with labor, as while women bear the family weight (mainly caring for the children) they will access part-time jobs to reconcile their work as mothers with that of workers. According to Ribeiro [64], the state will have to foster, through a social family policy, more egalitarian and fairer family structures, as well as creating contextual and legal conditions which respond to the new realities that support couples exercising their parental responsibilities compatibly with their professional work.

## 5. Material and Methods

Some studies apply econometric methodologies to model the likelihood of finding employment of the Spanish woman based on the personal characteristics of women (age, marital status, number of children, level of studies, family burdens, etc. [32,33,34,35,36,37,38]) using logit models, but there are no studies modeling the evolution of women’s employment through ARIMA models. This part of the paper, having examined women’s labor market to understanding how the labor market behaves, represents the originality of the research.

To know the evolution of the female labor market, the following types of information source were used:

- Information on the number of monthly working women (time series, from first quarter of 2008 to third quarter of 2020) [36].

The quantitative information was used to predict the potential demand of working women in Spain using an ARIMA model, based on a sample (51%) collected from the first quarter of 2008 to the third quarter of 2020, based on the Box–Jenkins (BJ) methodology [65]. According to Gujarati [66], the facilitating factor of this prediction method is in an analysis of the probabilistic, or stochastic, properties of the economic time series (in this case, the number of gastronomic tourists in Andalusia). In the time series models (BJ), the gastronomic tourist variable can be explained over time by its past or lag values and by the stochastic error terms, giving ARIMA models the advantage of being less costly in data collection, as only historical observations of the data are required.

According to published studies by Song and Li [67], the different versions of the ARIMA models proposed by Box and Jenkins [65] to identify, estimate, and diagnose dynamic models of time series have been applied in most post-2000 studies that used time series forecasting techniques. In the case of seasonal time series analysis, these models are called SARIMA, and they are differentiated from stationary ARIMA models in that the latter consider the mean of the series to be constant over time, and the correlation function depends on the lag and not the time at which it is calculated. However, the time series, in addition to random, cyclical, and seasonal variations, present a trend and seasonal components (the mean varies over time and by seasons), which makes the stationary processes unsuitable for modeling. For this reason, integrated models are introduced, thus eliminating the trend and seasonal component of these models.

The SARIMA models (p,d,q) × (P,D,Q)s are described by the following expressions:

φ (B) Φ(Bs) Zt = θ (B) Θ(Bs) at(1)

Zt = (1 − B)d (1 − Bs)D Yt (λ)(2)

where the operators introduced in the formulas are Yt (series observed, in our case, it is the gastronomic tourism demand), λ (the correction of the trend in variance of the series), Zt (series that is de-seasonalized and without a trend, that is stationary), B (lag operator), (1 − B) (typical difference operator), Bs (seasonal lag operator), and (1 − Bs) (seasonal difference operator). The difference operators and seasonal difference operators, in general, eliminate the trends and the seasonal components of the series, respectively. ϕ(B) is the autoregressive polynomial of order p, corresponding to the ordinary part of the series; θ(B) is the polynomial of moving averages of order q, corresponding to the ordinary part of the series; Φ(Bs) is the p-order autoregressive polynomial, corresponding to the seasonal part of the series; Θ(Bs) is the polynomial of moving averages of order Q, corresponding to the seasonal part of the series; at is the disturbance of the model; and D is the number of times the seasonal difference operators and typical difference are applied to the original series to make it stationary.

In the ARIMA and SARIMA models, the behavior of a time series is explained from the past observations of the series itself and the past forecasting errors. Several studies have shown how ARIMA models and their different variants obtain good results in forecasting.

## 6. Results

**Figure 6:**
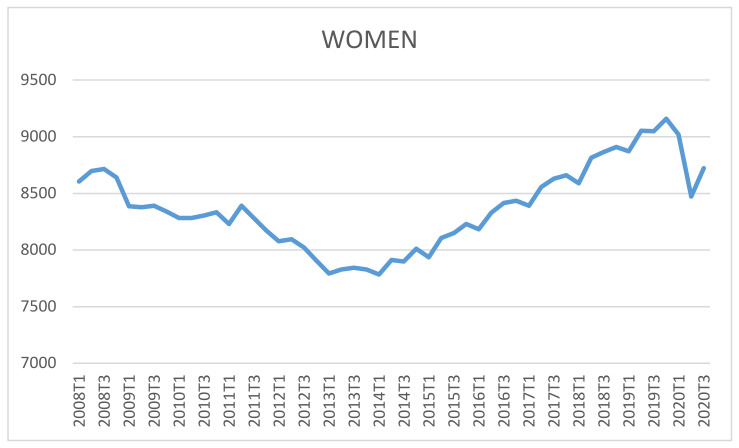
Evolution of female labor market. Source: Own elaboration from data of the Spanish National Statistics Institute (INE) [36].

Figure 6 shows a slightly growing trend for female labor market demand in the 12 years analyzed (first quarter of 2008 to third quarter of 2020). There was also variance, which was corrected with Box–Cox transformation, λ = 0.2; additionally, the mean trend and cycle trend were corrected by calculating the difference between the mean and cycle trends.

The SARIMA model [(1,1,1) (0,1,0)^12^ for the estimated female labor market was as follows (Table 7):

(1 − 0.3758057 B) (1 − B)^1^ (1 − B^12^)^1^ WOMEN^0.2 = (1 − 0.966704B) a_t_

t_ϕ1_ = 7.997565 *  t_θ1_ = −45.76333 *

* Significant parameters for α = 0.05 as can be seen by its probability (prob. column).

Table 8 and Table 9 show the different validation tests, which allow us to demonstrate that the model obtained is robust (Ljung–Box test for autocorrelation and ARCH test for heteroscedasticity, for the model). In the Ljung–Box test, the null hypothesis of absence of autocorrelation is met, i.e., the probability is higher than the 5% significance level (Prob. column).

The ARCH statistic (Table 9) indicates that, in the model, there is no autoregressive conditional heteroscedasticity (null hypothesis), i.e., the probability F (0.8644) is greater than the 5% significance level.

**Figure 7:**
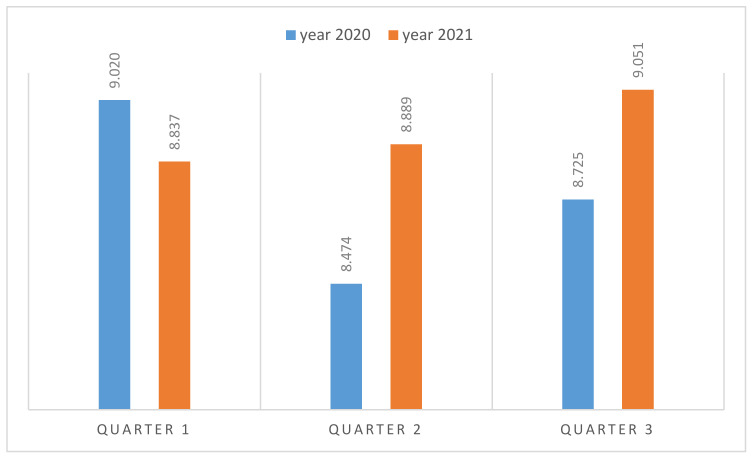
Prediction of the of female labor market (thousands of women) and its comparison with 2020). Source: own elaboration.

In Figure 7, the predictions obtained for 2021 and their comparison with 2020 are provided. The year 2020 has been atypical due to the pandemic, the closing of borders, and the restriction of movement within Spain. These predictions are made under the assumption that the pandemic is controlled and that the borders are fully open.

It is expected that, during 2021, there will be growth in the women’s labor market especially in the second quarter, if the COVID-19 vaccine is effective, demonstrating that the measures that are being implemented to increase the employability of the women’s collective are effective, although they can be improved.

## 7. Conclusions

The entry of Spanish women into the labor market is a phenomenon that began to be important from the 1960s as a consequence of a change of the society’s mentality. The male ceased being the only head of the family in charge of providing the income necessary to maintain it. Women can do this, especially in single-parent families. However, they still have not become aware of their empowerment. They feel guilty in part if they abandon the role of home carer for that of worker [68]. This also happens in other countries, as the research of Borelli [69] shows. To solve this problem, in addition to active employment policies, family policies which enable women to participate to a greater extent in all social areas, from politics to work, are needed.

As a consequence of a more open society, Spanish female workers have tried to train themselves and predominate in higher studies and degrees compared to males. All the same, the market has not corresponded to this increase of training.

This paper shows the presence of a glass ceiling that prevents women from reaching managerial occupations in Spain, a fact that occurs in other countries such as India [70].

The female unemployment rate is currently higher than that of males and the employment rate is lower, creating an imbalance in the labor market, which is also reflected in the precarious jobs that women do (similar results to those obtained by Campbell and Price [71]), even in sectors with a high rate of feminization, such as health and education. Women do not reach 10% of the number of hospital directors or university rectors in Spain. The stigma of the glass ceiling to access positions of responsibility, which is not only a Spanish problem but one at an international level [72], has to be eliminated through a greater awareness of society and through more active policies which foster equality: Measures which support the development of women in the child-bearing period are also needed, as their professional careers stay stuck in this period and it is difficult for them to rise to the next labor rung.

More stable jobs are also advocated (to increase the percentage of permanent contracts and complete working days) that will favor an increase of incomes in females, decreasing the existing wage gap. This problem is not exclusive to Spain, as noted by Kil et al. [73].

The more efficient way to reduce wage inequalities is to set a minimum wage which is the same for men and women. Moreover, according to Grout [74], offering the same promotion to men and women could be a good way to reduce consequently the difference between gender in high-rank positions. Increasing the number of women in managerial occupations through the introduction of quotas is not a good solution. Indeed, Rao [75] showed that, when quotas occur, the priority is to get women in order to fulfill the quota and it may be women who are not qualified.

The Spanish labor market is, to the expense of women, therefore ill adapted. Efficient and controlled measures that correct deviations in the short- and medium-term will allow a fairer and more egalitarian labor society for all groups.

Future lines of research include studying the women’s labor market according to the type of enterprises (public or private), in order to try to demonstrate that in the public sector there is less inequality than in the private sector in the women’s collective.

As limitations of this study is the lack of information on the part of private companies to provide anonymous information of their workers (salary, productivity, working hours, etc.), having had to resort to official data to carry out this investigation.

## Figures and Tables

**Table 1:** Comparison by sex of the main variables of the Spanish labor market.

		Year 2018	Year 2019	Difference 2019–2018
Employed (thousands)	Total	22,806.8	23,027.1	220.3▲
	Men	12,206.5	12,273.4	66.9▲
	Women	10,600.3	10,753.7	153.4▲
Unemployed (thousands)	Total	3479.1	3247.8	231.3▼
	Men	1674.6	1527.8	146.8▼
	Women	1804.5	1720.0	84.5▼
Employment rate (%)	Total	58.80	58.79	0.01▼
	Men	64.55	64.28	0.27▼
	Women	53.06	53.30	0.24▼
Unemployment rate (%)	Total	15.37	14.22	1.15▼
	Men	13.72	12.45	1.27▼
	Women	17.02	15.99	1.03▼

Source: Own elaboration from data of the Spanish National Statistics Institute (INE, (data free use)) [36]. ▲increase, ▼decrement.

**Table 2:** Indicators of monitoring and evaluation of the degree of fulfillment of the employment policy objectives.

Employment Policy Objectives	Indicators
1. Raise the participation in the work market and reduce the unemployment.	Main indicator
	–Employment rate 20–64
	Supplementary Indicators
	–Women’s employment rate
	–Youth employment rate
	–Employment rate of the over 45
	–Employment rate of the over 64
	–Unemployment rate 16–67
	–Youth employment rate
	–Unemployment rate of unskilled people
	–LTU 16–67
	–LTU youth
	–NEET (Young people not in education, employment, or training)
2. Reduce temporality and the segmentation of the labor market.	Main indicator
	–Temporary employment rate
	Supplementary Indicators
	–No. of promotion contracts
	–No. of transformations
	–No. of youth program contracts
3. Reinforce part-time work and firms’ internal flexibility.	Main indicator
	–Percentage of part-time workers
	Supplementary Indicators
	–No. of workers with reduced working day
	–Partiality and reduction in working hour rates, disaggregated by sex and age.
	–Evolution of the sickness rate
4. Improve and adapt the professional competences to the needs of the market.	Main indicators
	–School drop-out rate
	–Public spending on education (in % GDP)
	Supplementary Indicators
	–Population 30–34 years old with higher studies
	–Adult population in training for employment
5. Foster a fast and appropriate reintegration of people into the labor market.	Main indicators
	–Long-term unemployment rate
	–Investment in Active Employment Policies
	Supplementary Indicators
	–Long-term unemployment rates disaggregated by sex and age
6. Foster gender equality in the labor market.	Main indicators
	–Difference between employment rates of men and women
	–Difference between the average net wage per hour of men and women
	Supplementary Indicators
	–Difference between employment rates of men and women disaggregated by sex and age

Source: Ministry of Labor.

**Table 3:** Employed by type of contract according to sex, in thousands of people (fourth quarter of 2020).

Sector	Permanent Contract Men PCM	Permanent Contract Women PCW	Difference PCM-PCW	Temporary Contract Men TCM	Temporary Contract Women TCW	Difference TCM-TCW
Agriculture	199.3 *52.99%*	30.7 *39.66%*	168.6 *13.33%*	176.8 *47%*	46.7 *60.33%*	130.1 *−13.33%*
Industry	1506.5 *84.67%*	538.8 *80.53%*	967.7 *4.14%*	272.7 *15.33%*	130.2 *19.46%*	142.5 *−4.13%*
Construction	505.5 *59.35%*	73.4 *83.78%*	432.1 *−24.43%*	346.1 *40.64%*	14.2 *16.24%*	331.9 *24.4%*
Services	4.313.8 *79.13%*	5046.4 *74.04%*	−732.6 *5.09%*	1137.6 *20.86%*	1769.2 *25.95%*	−631.6 *−5.09%*

In italics is the percentage of type of contract of the sexes by sector of activity (e.g., in the agriculture sector, the permanent contracts of the female workers were 32.89% compared to 67.11% of the temporary contracts in the same sector, the sum of the sector being 100%). Source: Own elaboration from data of the Spanish National Statistics Institute (INE) [36].

**Table 4:** Unemployment rate by level of study and sex, in percentage (2020).

Studies Completed	Men	Women	Difference Women-Men
Illiterate	26.64	58.91	32.27
Primary studies not completed	26.95	47.50	20.55
Primary education	25.40	33.84	8.44
First stage of secondary education and similar	17.76	25.71	7.95
Second stage of secondary education with general orientation	15.04	21.13	6.09
Second stage of secondary education with professional orientation (including non-higher post-secondary education)	13.75	18.01	4.26
Higher education	9.70	12.44	2.74

Source: Own elaboration from data of the Spanish National Statistics Institute (INE) [36].

**Table 5:** Employed by sex and level of study (thousands of people) (2020).

Studies Completed	2020 T3			2020T 2			2020 T1		
	Total	Men	Women	Total	Men	Women	Total	Men	Women
Illiterate	37.3	23.3	14.0	38.2	24.5	13.7	45.0	27.3	17.7
Primary studies not completed	147.9	107.8	40.1	144.4	99.3	45.1	156.0	101.9	54.0
Primary education	699.1	437.7	261.4	727.8	458.3	269.4	827.5	520.6	306.9
First stage of secondary education and similar	4910.5	3144.6	1765.9	4786.2	3036.4	1749.9	5226.5	3275.8	1950.7
Second stage of secondary education with general orientation	2635.7	1442.7	1193.0	2483.0	1358.7	1124.4	2703.6	1466.0	1237.6
Second stage of secondary education with professional orientation (including non-higher post-secondary education)	1999.5	1086.5	913.0	1864.7	1009.1	855.7	1953.4	1045.3	908.1
Higher education	8746.8	4211.5	4535.3	8562.9	4147.2	4415.7	8769.2	4224.3	4545.0

Source: Own elaboration from data of the Spanish National Statistics Institute (INE) [36].

**Table 6:** Employment by sectors and sex, in percentage (third quarter of 2020).

Sector	Men (%)	Women	Difference Men-Women
A Agriculture, livestock farming, forestry, and fishing	5.9	2.1	3.8
B Extractive industries	0.2	0.1	0.1
C Manufacturing industry	15.3	6.8	8.5
D Supply of electricity, gas, vapor, and air conditioning	0.5	0.3	0.2
E Supply of water, sanitation activities, residue management and decontamination	1.1	0.3	0.8
F Construction	10.6	1.1	9.5
G Wholesale and retail business; repair of motor vehicles and motorcycles	13.3	15.6	−2.3
H Transport and storage	7.0	2.1	4.9
I Hospitality industry	7.2	9.0	−1.8
J Information and communications	3.7	1.9	1.8
K Financial and insurance activities	1.8	2.3	−0.5
L Real estate activities	0.6	0.9	−0.3
M Professional, scientific, and technical activities	4.3	5.1	−0.8
N Administrative activities and ancillary services	4.3	5.7	−1.4
O Public Administration and defense; Obligatory Social Security	7.0	6.1	0.9
P Education	3.8	8.1	−4.3
Q Health activities and social services	3.6	13.6	−10
R Artistic, leisure and entertainment activities	2.2	1.9	0.3
S Other services	1.4	3.4	−2
T Home activities as employees of domestic staff and producers of goods and services for own use	0.6	2.3	−1.7
U Activities of extraterritorial organizations and organisms	0.0	0.0	0

**Table 7:** Estimation of the demand for gastronomic tourism in Andalusia.

Dependent Variable: D(WOMEN^0.2,1,4)				
Method: Least Squares				
Variable	Coefficient	Std. Error	t-Statistic	Prob.
AR(1)	0.758057	0.094786	7.997565	0.0000
MA(1)	−0.966704	0.021124	−45.76333	0.0000

Source: Own elaboration.

**Table 8:** Ljung–Box statistics.

Sample: 2008Q1 2020Q3						
Autocorrelation	Partial Correlation		AC	PAC	Q-Stat	Prob
. | . |	. | . |	1	−0.051	−0.051	0.1226	
.*| . |	.*| . |	2	−0.069	−0.072	0.3558	
. |*. |	. |*. |	3	0.203	0.197	2.4376	0.118
.*| . |	. | . |	4	−0.067	−0.056	2.6705	0.263
. | . |	. | . |	5	−0.012	0.011	2.6777	0.444
. |*. |	. |*. |	6	0.118	0.074	3.4389	0.487
. | . |	. | . |	7	0.019	0.052	3.4597	0.629
.*| . |	.*| . |	8	−0.084	−0.078	3.8667	0.695
. |*. |	. | . |	9	0.095	0.062	4.3976	0.733
. | . |	. | . |	10	−0.050	−0.061	4.5514	0.804
.*| . |	. | . |	11	−0.066	−0.028	4.8202	0.850
. | . |	.*| . |	12	−0.025	−0.087	4.8592	0.900
. | . |	. | . |	13	−0.044	−0.030	4.9875	0.932
. | . |	. | . |	14	0.017	0.031	5.0078	0.958
.*| . |	.*| . |	15	−0.081	−0.081	5.4706	0.963
. | . |	. | . |	16	0.004	0.009	5.4721	0.978
.*| . |	.*| . |	17	−0.083	−0.088	5.9920	0.980
. | . |	. | . |	18	−0.047	−0.017	6.1633	0.986
.*| . |	.*| . |	19	−0.078	−0.103	6.6526	0.988
.*| . |	. | . |	20	−0.079	−0.065	7.1861	0.988

Source: Own elaboration. The * represent the values of the autocorrelation functions (AC) and Partial correlation.

**Table 9:** ARCH test for heteroscedasticity. Heteroskedasticity Test: ARCH.

Heteroskedasticity Test: ARCH			Prob.
F-statistic	0.027856	Prob. F(1,42)	0.8682
Obs × R-squared	0.029163	Prob. Chi-Square(1)	0.8644

Source: own elaboration.

## Data Availability

The data presented in this study are available on request from the corresponding author.
